# Parenting, Pesticides and Adolescent Psychological Adjustment: A Brief Report

**DOI:** 10.3390/ijerph19010540

**Published:** 2022-01-04

**Authors:** Joseph G. Grzywacz, Jason B. Belden, Amy M. Robertson, Daphne C. Hernandez, Fiorella L. Carlos Chavez, Michael J. Merten

**Affiliations:** 1 Department of Human Development and Family Sciences, Florida State University, Tallahassee, FL 32306, USA; amsmith3@fsu.edu; 2 Department of Integrative Biology, Oklahoma State University, Stillwater, OK 74078, USA; jbelden@okstate.edu; 3 Cizik School of Nursing, University of Texas Health Science Center at Houston, Houston, TX 77030, USA; Daphne.Hernandez@uth.tmc.edu; 4 Edson College of Nursing and Health Innovation, Arizona State University, Phoenix, AZ 85004, USA; Fiorella.Carlos.Chavez@asu.edu; 5 Department of Child, Youth, and Family Studies, University of Nebraska, Lincoln, NE 68588, USA; Michael.merten@unl.edu

**Keywords:** pesticides, adolescent depression, immigrants

## Abstract

Pesticides used to control insects, such as pyrethroids, are neurotoxicants, yet adolescent researchers often overlook their potential role in adolescent psychological adjustment. This brief report is guided by bioecological theory and considers the possible independent and interactive effects of environmental pyrethroid pesticide exposure for adolescent depressive symptoms. Self-reported adolescent appraisals of the parent–child relationship and depressive symptoms were obtained from a convenience sample of impoverished, predominantly Latino urban youth (*n* = 44). Exposure to environmental pyrethroids was obtained from wipe samples using a standardized protocol. Parent–adolescent conflict was higher in households with bifenthrin than those without, and adolescent depressive symptoms were elevated in homes where cypermethrin was detected. In addition, the presence of bifenthrin in the home attenuated the protective effects of parental involvement on adolescent depressive symptoms. The current results suggest that adolescent mental health researchers must consider the synergistic combinations of adolescents’ environments’ physical and social features. Given the endemic presence of pesticides and their neurotoxic function, pesticide exposure may demand specific attention.

## 1. Introduction

Pesticides are endemic in the environment, albeit in low doses [1], and may pose a risk to socioemotional adjustment during childhood and adolescence. Several pesticides are neurotoxicants affecting the neurotransmitter system, which has been associated with internalizing and externalizing problems in older children and adolescents [2]. Pyrethroid pesticides are the most common household pesticide [3], and although considered less toxic than organophosphate pesticides, epidemiologic studies have associated them with symptoms of attention-deficit and hyperactivity disorder (ADHD) among children and adolescents [4].

Adolescent researchers frequently study features of the social environment, particularly the parent–child relationship. Specifically, substantial evidence indicates that elevated parent–adolescent conflict and lower levels of parental involvement in adolescents’ lives are associated with greater depressive symptoms [5,6] and greater risk of clinical depression [7]. However, despite specific theoretical motivation [8], adolescent researchers rarely consider possible interaction effects of the physical and social environments. Following the bioecological model of human development [8], we posit that adolescent emotional health results from proximal processes in the home environment, specifically parent–adolescent conflict and the level of parental involvement. Further, we hypothesize that the form and power of these proximal processes are affected by exposure to environmental pesticides, even in small doses, e.g., applications in the home to control insects such as spiders, fleas or flies (personal or by an exterminator), or residues in the food or water supply [9]. That is, many organophosphate and pyrethroid pesticides can alter or modulate cholinergic neurotransmission in various regions of the brain, including the hippocampus [10,11]. Therefore, we posit that altered or modulated brain activity from possible pesticide exposure may mitigate otherwise developmentally generative parenting practices.

## 2. Materials and Methods

The data for this study are from a multi-faceted study of households in impoverished neighborhoods of Tulsa, Oklahoma (N = 91 parent–youth dyads) undertaken throughout 2013. The study neighborhoods were predominantly occupied by recent immigrants with modest educational levels (see Table 1), wherein approximately 40% of households were food insecure [12]. In addition, housing stocks in the study neighborhoods were old and primarily renter-occupied. The current analysis focuses on a subsample of participants (*n* = 44 parent–youth dyads) who completed a toxicological portion “add-on” to the study. The subsample used in this analysis is demographically similar to participants in the parent study (Table 1).

### 2.1. Procedure

Adolescents completed a self-administered survey questionnaire in their language of choice (English or Spanish) using a laptop computer provided by the research team and supported by the Qualtrics system. Environmental wipe samples were obtained using an established procedure [13]. Specifically, wipe samples were obtained from three smooth surfaces: (1) in the kitchen, typically the refrigerator, (2) a countertop in the bathroom most typically used by the adolescent, and (3) in the adolescent’s bedroom. At each location, a 10 × 10 cm template was placed on the surface and swabbed with two pads presaturated with 70% isopropyl alcohol (Covidien Webcol Alcohol Preps, 9 cm^2^). The first pad was wiped across the grid horizontally until the area was thoroughly wiped, and the second pad was wiped vertically. This process was repeated at each location, and all wipes from a single house were sealed into a glass vial creating a single composite sample for the house (300 cm^2^ sample size). An Institutional Review Board with Federal Wide Assurance approved all recruitment and data collection procedures detailed elsewhere [14].

### 2.2. Measures

Dependent variable. The 33-item Mood and Feelings Questionnaire, Child version (MFQ-C) [15], assessed depressive symptoms. The scores for the items were summed. Higher scores suggested higher levels of depressive symptoms (α = 0.92).

Independent variables. Parent–adolescent conflict was assessed with 20 items from the parent–child difficulties checklist [16]. Items were scored and summed (α = 0.92) such that higher scores indicated greater parent–adolescent conflict. Parental involvement was assessed with seven items capturing how often the focal youth and their caregiver spent time together [17]. Scores were summed (α = 0.83) such that higher scores indicated greater parental involvement. Both parent–adolescent conflict and parental involvement were centered on the sample mean to minimize multicollinearity problems resulting from the creation of multiplicative interaction terms [18].

All three wipes from within the home were composited into a single glass vial and analyzed as a single sample. Samples were stored at 4 °C up to 72 h prior to extraction and analysis.

### 2.3. Chemical Analysis

Analytical-grade bifenthrin and cypermethrin (mixed isomers) were the highest available purity (>98%) from Sigma-Aldrich (St. Louis, MO, USA). Deuterated PAHs were purchased from Accustandard (New Haven, CT, USA) for internal standards. All solvents were analytical or pesticide grade and obtained from VWR International (Radnor, PA, USA).

Extraction and analysis of wipe samples were performed similarly to previously described [19]. In each vial, 20 mL of ethyl acetate was added then placed in a sonicator for 20 min to aid extraction, and 10 mL of the ethyl acetate was recovered and evaporated to 1 mL. Analysis of extracts for pesticides was performed by gas chromatography coupled with mass spectrometry (GC/MS; Agilent 5975c inert XL MSD (Palo Alto, CA, USA)) using electron ionization (70 eV) similarly to previously described [20]. Separation was achieved using a 30 m × 250 µm × 0.25 µm HP-5 capillary column (Agilent). The inlet set temperature was 290 °C using a splitless injection. The oven was programmed to start at 110 °C, hold for 2.0 min, ramp at 8 °C/min to 275 °C, ramp at 12 °C/min to 310 °C and hold for 4.0 min. The flow rate was 2.0 mL/min using helium gas as the carrier. The following quantitative and qualitative ions were monitored bifenthrin (181:165, 166), cypermethrin (163:165, 181), chrysene d12 (240), and perylene d12 (264). Internal calibration was performed using chrysene d12 for bifenthrin and perylene d12 for cypermethrin. Isomers of cypermethrin chromatographed as 4 peaks and the average concentration across the peaks was used for quantitation. Quantitation limits were set at the lowest level greater than 3× the method detection limit and represented the lowest concentration that could be consistently used in calibration curves. Quality control was performed by wiping treated stainless steel surfaces. To treat the surface, 10 µL aliquots of an analytical standard using acetone as a carrier were placed in 10 locations within a 10 cm area and evaporation was allowed for 20 min prior to wiping the surface (*n* = 4). Mean recoveries and relative standard deviations were 81 ± 11 and 108 ± 13 for bifenthrin and cypermethrin, respectively. Blank samples (surface wipes of untreated surfaces) were also conducted and below detection limits for all analytes (*n* = 4).

The GC/MS analyses returned results for several organophosphate and pyrethroid pesticides. However, the current manuscript focuses on two pyrethroid pesticides—bifenthrin and cypermethrin—because of suggestive previous human and animal studies [4,21]. Dichotomous indicators of any exposure were created for each pesticide such that samples with zero detectible levels of pesticide were coded zero. In contrast, detection levels greater than zero were coded one.

A stepwise ordinary least squares model was fit using SPSS version 25, wherein adolescent depressive symptoms were regressed on covariates. First, adolescent depressive symptoms were regressed on mean-centered adolescent-reported parental involvement and parent–adolescent conflict, controlling for adolescent ethnicity (Latino ethnicity, yes versus no) and adolescent gender (Step 1). Next, dichotomous variables reflecting detection of any bifenthrin and any cypermethrin, respectively, were entered in the second step of the regression model to determine if pesticide exposure was associated with adolescent depressive symptoms (Step 2). Finally, multiplicative interaction terms of each dichotomous exposure variable (i.e., bifenthrin and cypermethrin) with each mean-centered parenting variable (i.e., parental involvement and parent-adolescent interaction) were constructed and added in the final steps of the modeling strategy. All the interaction terms were not entered simultaneously to avoid overfitting the model. Instead, we first tested interactions of parental involvement with each pesticide exposure variable (Step 3) and then interactions of parent–adolescent conflict with each pesticide exposure variable (Step 4).

## 3. Results

The range of observed adolescent depressive symptoms in the toxicology subsample was from 32 to 62 (M = 43.8, SD = 7.4), which does not differ from levels of depressive symptoms reported for total sample [13], t(42) = −0.65, *p* = 0.520. Depressive symptoms had a moderate skew of 0.6 (SE = 0.4), although the Shapiro–Wilk test indicated normality (W = 0.95, *p* > 0.05). Greater adolescent depressive symptoms were associated with more parent–adolescent conflict (r = 0.39, *p* < 0.01) and less parental involvement (r = −0.38, *p* < 0.05).

Focal pesticides were detected at trace levels. The median level of bifenthrin was 38.7 ng/cm^2^, well below the sample’s 95% percentile (354.8 ng/cm^2^) and maximum value (3455.6 ng/cm^2^). Similarly, the median level of cypermethrin was 430.7 ng/cm^2^, again well below the sample’s 95% percentile (1995.3 ng/cm^2^) and the maximum value (2398.8 ng/cm^2^). Therefore, most households had trace levels of bifenthrin or cypermethrin.

Cypermethrin was detected in 32% of the households, while bifenthrin was detected in 23% of the households. Parent–adolescent conflict was higher in households with bifenthrin than those without (M = 69.4, SD = 27.4 and M = 52.4, SD = 17.2, respectively; t = −2.37, df = 41, *p* < 0.05), and adolescent depressive symptoms were elevated in households where cypermethrin was detected relative to households without this pesticide (M = 47.9, SD = 7.0 and 42.0, SD = 6.9, respectively; t = −2.57, df = 41, *p* < 0.05).

Results obtained from linear regression models indicated that higher levels of parent–adolescent involvement were associated with fewer adolescent depressive symptoms. In comparison, higher levels of parent–adolescent conflict were associated with greater depressive symptoms in the first step (Table 2). Only the association of parent–adolescent conflict with depressive symptoms remained after pesticide exposure was added to the model in step two, with a resulting R^2^ increase of 0.10. Detection of cypermethrin in the household was associated with greater depressive symptoms, with the observed association having an f^2^ value of 0.11 indicative of “small” effect size. In the third step, parent–adolescent involvement was interacted with each pesticide exposure variable, and the interaction with bifenthrin exposure was independently associated with depressive symptoms. Parental involvement was associated with fewer depressive symptoms in households where bifenthrin was not detected. However, in homes where bifenthrin was seen, the beneficial effect of parental involvement for depressive symptoms was attenuated. The observed interaction increased R^2^ from 0.39 to 0.54, reflecting a medium-sized effect (f^2^ = 0.18). The interactions between cypermethrin and the parent–child relationship variables were not statistically significant.

## 4. Discussion

The results are consistent with fundamental theories of human development [8], the primary mechanism of action of the pesticide, and previous studies implicating pesticide exposure in emotional well-being or children’s psychological adjustment [22]. Nevertheless, we must acknowledge the limitations of this study, including the small sample and corresponding unstable parameter estimates. It is also essential to remind readers that most households had trace levels of pesticide detected in our wipe samples. It is also unclear if the observed correlations are based on causative effects of the pyrethroid insecticides or related to a secondary effect such as homes requiring pest control. Research indicates elevated depressive symptoms in housing units with cockroach and mouse infestations [23]. Therefore, the results of this study should be interpreted as exploratory and with caution.

Nevertheless, there was a clear signal in these data suggesting the possibility that cumulative low-dose exposure to cypermethrin may compound over time to undermine psychological adjustment. More striking is the new finding that bifenthrin may attenuate the protective effects of parental involvement, a widely recognized “protective factor” for children and adolescents. Minimally, these results draw needed attention to both the physical and social attributes of the adolescent exposome [24] and how they may contribute to systematic variation in adolescent emotional health and psychological adjustment. Future research is needed to replicate these findings. Importantly, additional studies with larger samples (to stabilize parameter estimates), other environmental assessments perhaps from the air or dust sampling (to minimize misclassification of exposure), and possibly biomarkers of adolescent pesticide exposure are needed. Nevertheless, the current findings have potentially substantial implications for future research on adolescent emotional health and psychosocial development if replicated.

## 5. Conclusions

Pesticides are endemic in society, and these results suggest that even trace exposure to pyrethroid insecticide in the home is associated with levels of depressive symptoms among adolescents. Moreover, pyrethroid exposure also attenuated the protective effects of parental involvement. If replicated, research on adolescent development may need to give greater attention to pesticides.

## Figures and Tables

**Table 1:** Sample description of the parent study and the subsample who participated in the toxicological portion of the study.

	Total Sample (N = 91)		Toxicology Sub-Sample (*n* = 44)	
	%	M (SD)	%	M (SD)
Parent				
Age		39 (6.3)		39 (5.8)
Gender (female = 1)	89.2		92.2	
Race (White = 1)	85.9		82.4	
Ethnicity (Latino = 1)	78.9		78.4	
Education				
Did not graduate HS	54.3		52.9	
HS. Graduate	20.7		21.6	
Marital Status				
Currently Married	54.9		52.9	
Living as Married	23.1		17.6	
Adolescent				
Age		14 (1.9)		14.4 (1.7)
Gender (female = 1)	45.7		40.0	
Race (White = 1)	81.5		78.0	
Ethnicity (Latino = 1)	80.0		79.6	
Education				
5–6th grade	11.0		7.1	
7–8th grade	27.5		30.9	
9–12th grade	61.6		62.0	

**Table 2:** Ordinary least squares standardized regression results from models of the number of adolescent depressive symptoms.

	Model 1 *β*	Model 2 *β*	Model 3 *β*	Model 4 *β*
*Step 1*				
Parent-Adolescent Involvement	−0.37 *	−0.27	−0.66 **	−0.22
Parent-Adolescent Conflict	0.35 *	0.39 *	0.32 *	0.54 *
*Step 2*				
Bifenthrin		−0.06	−0.29	−0.01
Cypermethrin		0.32 *	0.28 *	0.36 *
*Step 3*				
Parent-Adolescent Involvement X Bifenthrin			0.48 **	-
Parent-Adolescent Involvement X Cypermethrin			0.35	-
*Step 4*				
Parent-Adolescent Conflict X Bifenthrin			-	−0.31
Parent-Adolescent Conflict X Cypermethrin			-	0.11
R^2^	0.29	0.39	0.54	0.43
ΔR^2^	0.29	0.10	0.15	−0.11

Note: all models control for the effects of adolescent ethnicity (Latino, yes versus no) and adolescent gender. * *p* < 0.05, ** *p* < 0.01.

## Data Availability

The data presented in this study are available on request from the corresponding author. The data are not publicly available due to constraints in the original informed consent which did not indicate that de-identified information would be shared with other researchers.
